# Exploring structural barriers to diabetes self-management in Alberta First Nations communities

**DOI:** 10.1186/s13098-018-0385-7

**Published:** 2018-12-03

**Authors:** Stephanie Kulhawy-Wibe, Kathryn M. King-Shier, Cheryl Barnabe, Braden J. Manns, Brenda R. Hemmelgarn, David J. T. Campbell

**Affiliations:** 1 Department of Medicine, Cumming School of Medicine, University of Calgary, North Tower, 9th Floor, 3330 Hospital Drive NW, Calgary, AB T2N 4N1 Canada; 2 Department of Community Health Sciences, Cumming School of Medicine, O’Brien Institute for Public Health, Libin Cardiovascular Institute of Alberta, Faculty of Nursing, University of Calgary, TRW 3rd Floor, 3330 Hospital Drive NW, Calgary, AB T2N 4N1 Canada; 3 Departments of Medicine and Community Health Sciences, Cumming School of Medicine, O’Brien Institute for Public Health, Libin Cardiovascular Institute of Alberta, University of Calgary, North Tower, 9th Floor, 3330 Hospital Drive NW, Calgary, AB T2N 4N1 Canada; 4 Department of Medicine, Cumming School of Medicine, University of Calgary, 1820 Richmond Road SW, Calgary, AB T2N 4N1 Canada

**Keywords:** Diabetes mellitus, First Nations, Barriers to care, Access to care, Diabetes education, Financial barriers

## Abstract

**Background:**

Type 2 diabetes is highly prevalent in Canadian First Nations (FN) communities. FN individuals with diabetes are less likely to receive guideline recommended care and access specialist care. They are also less likely to be able to engage in optimal self-management behaviours. While the systemic and racial contributors to this problem have been well described, individuals’ experiences with structural barriers to care and self-management remain under-characterized.

**Methods:**

We utilized qualitative methods to gain insight into the structural barriers to self-management experienced by FN individuals with diabetes. We conducted a qualitative descriptive analysis of a subcohort of patients with diabetes from FN communities (n = 5) from a larger qualitative study. Using detailed semi-structured telephone interviews, we inquired about participants’ diabetes and barriers to diabetes self-management. Inductive thematic analysis was performed in duplicate using NVivo 10.

**Results:**

The structural barriers faced by this population were substantial yet distinct from those described by non-FN individuals with diabetes. For example, medication costs, which are usually cited as a barrier to care, are covered for FN persons with status. The barriers to diabetes self-management that were commonly experienced in this cohort included transportation-related difficulties, financial barriers to uninsured health services, and lack of accessible diabetes education and resultant knowledge gaps.

**Conclusions:**

FN Albertans with diabetes face a myriad of barriers to self-management, which are distinct from the Non-FN population. In addition to the barriers introduced by colonialism and historical injustices, finances, geographic isolation, and lack of diabetes education each impede optimal management of diabetes. Programs targeted at addressing FN-specific barriers may improve aspects of diabetes self-management in this population.

## Background

Diabetes mellitus (DM) is a consequence of colonization that remains a substantial problem for indigenous communities across Canada [1–3]. First Nations (FN) individuals have a 76–87% lifetime risk of developing diabetes [4]. Canadian indigenous populations shoulder a heavy disease burden as evidenced by the high incidence of diabetes [1, 5]. Additionally, FN individuals have higher rates of diabetes-related hospitalizations [6], chronic complications [7, 8], and mortality [9]. Many have articulated how health system deficits and historical injustices are to blame for these disparities in care and outcomes [10]. These deficits include discordance between patient and provider viewpoints [11], ineffective doctor-patient communication [12], mistrust of the healthcare system related to the Canadian legacy of residential schools [13, 14], and tuberculosis sanatoria [15]; which may result in disengagement from health services [16–19].

Given these disparities and the historical context in which they occur, there is a need to explore the health experiences of FN individuals with diabetes, and identify practices and processes that are required to meet the needs of individual patients and support self-management [20]. Engagement in DM education programs, has been shown to improve diabetes-related outcomes [21] and that self-management support programs are more effective when adapted to overcome barriers faced by specific populations [22]. Furthermore, glycemic control is closer to target when perceived barriers to self-care are low [23]. However, prior studies in FN populations with diabetes have focused on identifying gaps in diabetes management and outcomes [24] and provider perspectives about barriers to care [25, 26], leaving a relative knowledge gap on the specific barriers and experiences of FN individuals with diabetes, particularly as related to self-management behaviours.

Recently, Jacklin et al. [27] thoroughly explored health care experiences of Indigenous Canadians with diabetes, and identified key themes of the colonial legacy of healthcare, the perpetuation of inequalities, and structural barriers to care. Their conclusions centre on the importance of healthcare provider training to provide patient-centred care that is founded in cultural safety. Cultural safety has been defined as “an environment which is safe for people; where there is no assault, challenge or denial of their identity, of who they are and what they need. It is about shared respect, shared meaning, shared knowledge and experience, of learning together with dignity, and truly listening” [28]. Jacklin et al. point out that structural barriers were a key theme, but their analysis and discussion focus largely on the other two themes, which are of critical importance in improving health equity for indigenous Canadians [27]. This paper, however, will use a different dataset to focus on the third theme they describe: structural barriers to optimal diabetes self-management.

## Methods

### Study design

Studies investigating patients’ barriers often use quantitative methods [23, 24]. However, individual experiences such as barriers to diabetes self-management may be more richly explored using qualitative methods [29]. Therefore, we chose to use qualitative methods to explore and describe perspectives of FN individuals with regards to the barriers they face in the management of diabetes.

Our work was embedded within a larger qualitative study focused on financial barriers to care, where data was collected from 34 participants with cardiovascular-related chronic diseases, which included both FN and non-FN individuals [30]. Within this larger study, among those with diabetes, we found compelling differences in the experiences reported by participants living in FN communities and non-FN participants. We presented the principal financial barriers for non-FN participants in a separate publication [31]—in this manuscript we seek to highlight the structural barriers to effective diabetes self-management raised by our FN participants.

### Sampling and data collection

Full details of sampling and data collection are reported elsewhere [32]. Participants (n = 5) were recruited purposively [33] via signage in physician offices and pharmacies, clinical databases, and a FN kidney disease prevention clinic [34]. We collected data using semi-structured telephone interviews that focused on individuals’ experiences of diabetes and financial barriers to care. Interviews were digitally recorded. Recordings of interviews were transcribed verbatim by a professional transcriptionist. Ethics approval was received by the University of Calgary’s Conjoint Health Research Ethics Board. Informed consent was obtained verbally and recorded for all participants.

### Data analysis

In the broader qualitative study, we collected and analyzed data concurrently. This allowed us to continue sampling and data collection until saturation was achieved for the parent study (though not specifically for the FN subcohort). We undertook a secondary analysis of the data from FN participants. Transcribed data were imported into NVivo 10 software (QSR International: Doncaster, Australia) for analysis purposes. We used an inductive thematic analysis strategy to generate codes and themes from our recorded interviews [35]. All analyses were done in duplicate including one experienced qualitative researcher with formal training in health services research and anthropology (DC) and a clinical trainee (SKW). The analysis was supervised and supported by an established expert in qualitative research (KKS).

## Results

We explored the structural barriers to diabetes management experienced by 5 participants living in FN communities, who were interviewed as part of the larger study. This sample included four women and one man, between 30 and 65 years of age. All participants lived in First Nations communities adjacent to a major western Canadian city.

As described by Jacklin et al. [27], unique barriers exist for FN individuals with diabetes, sculpted by the complex history leading to policies that affect the daily lives of FN people today. Since that paper focused on the social and historical barriers, we have decided to focus on the financial and structural factors that complicate diabetes self-management. We grouped these reported barriers thematically into three major categories: geography; finances and health insurance; and lack of diabetes education. We describe these themes, supported with direct quotations (*italicised*) from the interviews and discuss each theme in the context of supporting literature.

### Geography

In Alberta, nearly 40% of all FN persons live in FN communities or reserves [36]. These are crown lands protected for the exclusive habitation and use of FN persons with registered status [37]. Unfortunately, as our respondents shared, the geographic isolation of these lands creates a physical barrier to accessing care services.

Transportation to an urban centre to access specialist and allied health care was cited as a significant barrier. While government-sponsored transportation services were reportedly available, participants felt that they were overly difficult to access, inconvenient, or inefficient. Some participants reported that transportation was only offered for appointments with physicians but not to see allied health providers: “*I still haven’t really dealt with the diabetes clinic because I keep getting denied for transportation”.*

Endeavours to arrange transportation outside of official options were discussed. Participants described that not having access to a personal vehicle posed a significant problem in seeking required care: “*I’ve never got my license so I wasn’t able to get a vehicle. And where I live it’s like you need a ride everywhere… I don’t have transportation here.”* Other informal transportation arrangements often required personal payment and were inflexible, relying on another person’s availability. Even among those who had personal vehicles, the costs of gas, parking, and other transportation-related expenses were significant barriers: “*I would have to budget at least $200 a month for gas going to the city”.*

Beyond contributing to difficulties accessing care, geographic considerations also contributed to difficulties in diet adherence. Appropriate dietary intake, including fresh fruits and vegetables, low glycemic index foods and lean sources of protein, is central to diabetes self-management [38]. Yet, our participants reported significant difficulties accessing appropriate healthy food. This problem was multi-faceted, but certainly exacerbated by geographic isolation. One participant identified that the food available in the community was not ideal: “*It was just the distance, eh? Having to go out and getting the fresh food. There is a store here but their food is not always fresh”.* The lack of availability of fresh fruits and vegetables in local communities necessitated travelling to purchase these staples from larger full-service grocery stores:

*We don’t have the big stores here to go buy fruit and vegetables all the time. We have to travel half an hour to an hour out of the community to go buy milk, dairy products, the good food they advise us to, we weren’t prone to having those. So much of our diet includes bannock, potatoes, meat, a lot of meat and very little of vegetables and fruit.*

Without convenient local food sources, frequent trips to the grocery store become near impossible, making it easier to stock up on processed foods rather than to buy fresh foods that may spoil: *“The healthy foods seem to spoil easily. So that’s a challenge for me because I’m used to just getting things that don’t spoil. Just the fast foods I guess, just instant”.*

### Finances and health insurance

FN persons with Treaty Status (including 83% of FN people in Alberta [36]) have prescription medication insurance coverage (with no co-payment or deductible) through the Non-Insured Health Benefits program (NIHB), administered by the First Nations and Inuit Health Branch of Health Canada [39]. For qualified beneficiaries, NIHB is the payer for a limited set of pharmacy, transportation, medical supplies and equipment, dental and vision care. For drug benefits, the dispensing pharmacy submits a claim to the program directly meaning that no point of dispensation charges are required of patients who display their identification card. Given this arrangement, the cost of medications was less problematic for study participants compared to the situation for non-FN persons with diabetes [31, 32, 40]: “*I’m treaty status and so far non*-*insured health benefits which is a Department of Indian Affairs, or Aboriginal Affairs or whatever they call it today. So I get coverage every month for all my supplies and the insulin that I’m on”.*

Despite full medication coverage, the remaining costs associated with diabetes self-management were often prohibitive for participants. Obtaining diabetes supplies, such as blood glucose testing strips, was financially difficult for most participants despite partial public funding through NIHB. The amount covered was often deemed to be insufficient to meet the perceived testing needs of several of our participants. One participant explained: “*They weren’t prescribing enough of the supplies for me… I was running out and I don’t wanna spend like 70 extra dollars every month.”* Some participants described having to resort to cost-saving strategies such as reusing needles and lancets, which may predispose to infectious complications.

Diabetes testing and injection supplies were not the only aspect of care to which FN individuals experienced financial barriers. All study participants reported a prohibitive financial barrier to accessing at least one of dental, vision, or foot care related to NIHB policies and limited coverage. These allied health services are especially important in the context of diabetes as they help detect and care for end organ damage that results from diabetic microvascular complications. For example, persons with diabetes require vigilant foot care and ulcer management as the disease is characterized by poor wound healing as well as an increased likelihood of acquiring foot ulcers, with a propensity for developing infections [41]. One participant described the struggle to access foot care: “*Like I said, my feet, that’s what I really wanna take care of but we can’t afford it yet. It’s just when I have spare money.”* Similarly, another microvascular complication of diabetes is diabetic retinopathy [42], additionally, macular edema, cataracts, glaucoma [43] and simple myopia [44] leading to impaired vision [45] are all much more prevalent among patients with diabetes. This participant found meeting his vision needs was financially difficult: *“And the biggest problem I find is I needed new glasses not too long ago and the government won’t pay for them.”*

Finally, in addition to the geographic barriers to eating healthy, described above, many participants described a significant financial cost associated with healthy food: *“It’s something that I always need and they get pricey, buying a lot of fruit and vegetables… all the junk food is cheap”*. It became clear through our interviews, that some are simply overwhelmed by the challenge of trying to obtain healthy food while balancing their many other financial demands:

*Like, the vegetables and the fruit and that are more expensive now… And like for me I have to feed 7 of us now. My granddaughter’s here. And I’m the only one with the income… It’s kinda hard financially to do all the travel, the food, the bills. That’s where it’s hard for me in the, and then the food’s getting so expensive now.*

### Lack of quality diabetes education

Despite diabetes education services being offered in many communities, some participants experienced a lack of ready access to high quality diabetes education: *“I’ve only met the diabetes nurse just once and she doesn’t really seem helpful.”* Specifically, several participants were not aware of or were unsatisfied with diabetes education offered in the community, but were willing to access education in the tertiary care setting, which would necessitate transportation to resources outside the community: *“But I wish they had more programs for that ‘cause I wanted to try to see if I can go to [town] if they had any evening courses.”*

Due to a perceived lack of availability of reliable diabetes education in the community, some participants turned to the Internet: “*I haven’t been able to see a dietician yet for diabetes. So to me I think that’s important because, I’m figuring things out on my own through the internet. And I think these people would be more useful for me than the computer.”* Ultimately, using the internet lacked the personal and relational support that could have been provided in a face to face meeting.

This lack of diabetes education seemed to lead to knowledge gaps about all aspects of diabetes, from prevention/etiology to treatment (including health behaviours). One participant stated: *“I was originally on the insulin pen because my sugar was really high. When I went in it was at 38 and I didn’t know that the normal is 7.”* Some participants described that diabetes was completely unfamiliar: “*No one in my family is diabetic and so it was new to me.”* Knowledge gaps also limited patients’ ability to manage their condition and make positive health behaviour change. About dietary changes, one participant stated: “*I don’t know how to read these boxes… I just really haven’t learned how to eat, so basically I just eat what I usually eat. We’re just used to eating the same old thing, for me, it’s just what I grew up with,”*

These knowledge gaps created stress that seemed to weigh heavily on the minds of those we interviewed. For many, this stress centered on wanting to do something to help themselves, but being paralyzed by not knowing what to do or not feeling empowered to act on knowledge they did have: *“It’s always very close to my mind, what we can do and what I can do. I mean certainly my health leads to this or that but what can I, where can I go, what do I need to do?”* Some voiced a degree of fatalism, that poor outcomes were an inevitability: *“I just thought that it was a death sentence to be diabetic.”*

A lack of diabetes education and poor diabetes knowledge is associated with a greater degree of diabetes-related stigma [46]. This was certainly the case with our participants who described having a hard time admitting or accepting that they were diabetic or felt embarrassed about their diagnosis. *“When I got it I was a young person and I quite resented it because I was different from all the other kids… it was sort of ‘what did I do wrong to get diabetes?’”* Many struggled with receiving a diagnosis, with several identifying themselves as a “sick person”. Some voiced a belief that diabetes was somehow a punishment, or preventable, and thus there seemed to be an element of guilt and shame that came with the diagnosis: *“Guilt… is probably the biggest feeling that I’ve had over the last few years. Is that it’s a punishment because I’m not healthy and maybe I didn’t make the right choices in my youth or whatever.”*

## Discussion

Diabetes is a growing problem in FN communities across Canada [1–3] and worse yet is that this population continues to be ineffectually served by the current health system leading to poor disease outcomes [10, 16–18]. Barriers to optimal care are complex and inextricably intertwined with culture, values, history, and geography. Jacklin et al. [27] recently examined the role of healthcare provider reeducation/training to improve patient centered care and promote cultural safety in consideration of the colonial legacy of healthcare and present day perpetuation of inequalities. Here, we explore the unique structural barriers to diabetes self-management experienced by FN patients.

Finances were a major concern in one form or another for all participants. FN Canadians are known to have a significantly lower total annual income compared with non-FN Canadians (CAD $19,114 compared with CAD $33,394) [36, 47]. Many of these financial barriers were exaggerated by the geographic isolation that is faced by many FN individuals. Our findings about geographic isolation were similar to other studies suggesting that geographic isolation is not entirely responsible for poor health outcomes, but is a significant contributor when coupled with low socioeconomic status and historical factors [48, 49]. In addition, we found that quality diabetes education was lacking, which hinders diabetes self-management and promotes disease stigma. This too was limited by geographic isolation, as many of the programs available are offered in major urban centres. This has been addressed in Australian Aboriginal populations with online resources and has been shown to improve diabetes outcomes [50].

The barriers reported in this study are likely more complex and deep rooted than the individual proximal negative experiences that we heard. Undoubtedly, generations of mistreatment and multigenerational trauma contribute significantly to these difficulties, as recently explored by the Truth and Reconciliation Committee [14], and the recent paper by Jacklin et al. [27]. The failed historical relations between government and FN communities limit health care delivery to FN individuals. This history, as well as ongoing racism and exclusion, leads to considerable mistrust of healthcare providers that limits the quality of care that can be provided. Care providers must be mindful of these issues, and practice cultural humility [51, 52] with openness to Indigenous ways of understanding health [53] that incorporate patient centered interventions [26] in order to produce productive therapeutic relationships [27]. Furthermore, Hackett [19] suggests that acknowledging the failed historical past of the healthcare system’s interactions with FN communities could be incorporated into educational programs to break down the legacy of mistrust.

## Limitations

This exploratory study is limited to the perspectives of the individuals interviewed. The sample contributing to this data is quite small, even for typical qualitative research. Morse [54] has suggested that for phenomenological studies, small samples of 6–10 can be adequate. Phenomenology is a methodology used to understand participants’ lived experiences [55], while we did not undertake true phenomenology, this work clearly followed some phenomenological principles-attempting to understand and characterize the experience of structural barriers in the context of diabetes for FN Canadians. Furthermore, qualitative studies, which ask a focused question of a homogenous group require smaller samples [33]. While in the broader qualitative study, participants were sampled to saturation; the data obtained from this sub cohort was not fully saturated. Despite these limitations, we feel that the narratives in this study undeniably elucidate real barriers that may well be experienced more broadly by many FN individuals with diabetes in Canada. Others have written that: “a case study involving a single research participant can be of importance and can generate great insight. This logically means that the smallest acceptable sample size in these types of qualitative research is a sample of one” [56].

Future research in this area should incorporate indigenous ways of knowing [57] and participatory-action methods [58] within an Aboriginal-guided research framework [59] to achieve a better understanding of the structural barriers faced by FN individuals with diabetes. It would also be helpful to further quantify the presence of various types of financial barriers for FN individuals with status and without status, as well as FN peoples who live in FN communities compared to those who live in major urban centres.

## Conclusions

Through our interviews, it became evident that diabetes care for persons living in First Nations communities is a complex issue with a myriad of barriers—which include structural factors. Many of the important cost-related barriers were exacerbated by geographic isolation. These included the direct and indirect costs associated with travelling into the city for specialist appointments, allied health care, and to obtain healthy food. Using in-depth qualitative methods, we have gained a greater understanding of FN patients’ experiences with barriers to diabetes care and propose several avenues for future research and policy that may help achieve better health outcomes for First Nations persons with diabetes.

## Abbreviations

DM: diabetes mellitus
FN: First Nations
NIHB: Non-Insured Health Benefits program

## References

[1] Dyck R, Osgood N, Lin TH, Gao A, Stang MR (2010). Epidemiology of diabetes mellitus among First Nations and non-First Nations adults. Can Med Assoc J.

[2] Riediger ND, Lix LM, Lukianchuk V, Bruce S (2014). Trends in diabetes and cardiometabolic conditions in a Canadian First Nation community, 2002–2003 to 2011–2012. Prev Chronic Dis..

[3] Johnson JA, Vermeulen SU, Toth EL, Hemmelgarn BR, Ralph-Campbell K, Hugel G (2009). Increasing incidence and prevalence of diabetes among the Status Aboriginal population in urban and rural Alberta, 1995–2006. Can J Public Health.

[4] Turin TC, Saad N, Jun M, Tonelli M, Ma Z, Barnabe CC (2016). Lifetime risk of diabetes among First Nations and non-First Nations people. Can Med Assoc J.

[5] Yeates K, Tonelli M (2006). Indigenous health: update on the impact of diabetes and chronic kidney disease. Curr Opin Nephrol Hypertens.

[6] Campbell DJ, Lacny SL, Weaver RG, Manns BJ, Tonelli M, Barnabe C, Hemmelgarn BR (2014). Age modification of diabetes-related hospitalization among First Nations adults in Alberta, Canada. Diabetol Metab Syndr..

[7] Samuel SM, Palacios-Derflingher L, Tonelli M, Manns B, Crowshoe L, Ahmed SB (2014). Association between First Nations ethnicity and progression to kidney failure by presence and severity of albuminuria. Can Med Assoc J.

[8] Jiang Y, Osgood N, Lim HJ, Stang MR, Dyck R (2014). Differential mortality and the excess burden of end-stage renal disease among First Nations people with diabetes mellitus: a competing-risks analysis. Can Med Assoc J.

[9] Park J, Tjepkema M, Goedhuis N, Pennock J (2015). Avoidable mortality among First Nations adults in Canada: a cohort analysis. Health Rep.

[10] Marrone S (2007). Understanding barriers to health care: a review of disparities in health care services among indigenous populations. Int J Circumpolar Health..

[11] Thurston WE, Coupal S, Jones CA, Crowshoe LF, Marshall DA, Homik J (2014). Discordant indigenous and provider frames explain challenges in improving access to arthritis care: a qualitative study using constructivist grounded theory. Int J Equity Health..

[12] Towle A, Godolphin W, Alexander T (2006). Doctor-patient communications in the Aboriginal community: towards the development of educational programs. Patient Educ Couns.

[13] MacDonald DB, Hudson G (2012). The genocide question and Indian residential schools in Canada. Can J Polit Sci.

[14] Truth and Reconciliation Commission of Canada (2015). Honouring the truth, reconciling for the future: Summary of the final report of the Truth and Reconciliation Commission of Canada.

[15] Hidgson C (1982). The social and political implications of tuberculosis among native Canadians. Can Rev Sociol Anthropol..

[16] Deved V, Jette N, Quan H, Tonelli M, Manns B, Soo A (2013). Quality of care for First Nations and non-First Nations People with diabetes. Clin J Am Soc Nephrol.

[17] Gao S, Manns BJ, Culleton BF, Tonelli M, Quan H, Crowshoe L (2008). Access to health care among status Aboriginal people with chronic kidney disease. CMAJ.

[18] Campbell DJ, Ronksley PE, Hemmelgarn BR, Zhang J, Barnabe C, Tonelli M (2012). Association of enrolment in primary care networks with diabetes care and outcomes among First Nations and low-income Albertans. Open Med..

[19] Hackett P (2005). From past to present: understanding First Nations health patterns in a historical context. Can J Public Health.

[20] Ka Rice (2016). Best practices for the prevention and management of diabetes and obesity-related chronic disease among indigenous peoples in Canada: a review. Can J Diabetes..

[21] Chrvala CA, Sherr D, Lipman RD (2016). Diabetes self-management education for adults with type 2 diabetes mellitus: a systematic review of the effect on glycemic control. Patient Educ Couns.

[22] Hill-Briggs F, Lazo M, Peyrot M, Doswell A, Chang YT, Hill MN (2011). Effect of problem-solving-based diabetes self-management training on diabetes control in a low income patient sample. J Gen Intern Med.

[23] Daniel M, Messer LC (2002). Perceptions of disease severity and barriers to self-care predict glycemic control in Aboriginal persons with type 2 diabetes mellitus. Chronic Dis Can..

[24] Harris SB, Naqshbandi M, Bhattacharyya O, Hanley AJ, Esler JG, Zinman B (2011). Major gaps in diabetes clinical care among Canada’s First Nations: results of the CIRCLE study. Diabetes Res Clin Pract.

[25] Bhattacharyya OK, Rasooly IR, Naqshbandi M, Estey EA, Esler J, Toth E (2011). Challenges to the provision of diabetes care in first nations communities: results from a national survey of healthcare providers in Canada. BMC Health Serv Res..

[26] Bhattacharyya OK, Estey EA, Rasooly IR, Harris S, Zwarenstein M, Barnsley J (2011). Providers’ perceptions of barriers to the management of type 2 diabetes in remote Aboriginal settings. Int J Circumpolar Health..

[27] Jacklin KM, Henderson RI, Green ME, Walker LM, Calam B, Crowshoe LJ (2017). Health care experiences of Indigenous people living with type 2 diabetes in Canada. Can Med Assoc J.

[28] Williams R (1999). Cultural safety—what does it mean for our work practice?. Aust N Z J Public Health.

[29] Ritholz MD, Beverly EA, Weinger K (2011). Digging deeper: the role of qualitative research in behavioral diabetes. Curr Diab Rep..

[30] Campbell DJ, Manns BJ, Leblanc P, Hemmelgarn BR, Sanmartin C, King-Shier K (2016). Finding resiliency in the face of financial barriers: development of a conceptual framework for people with cardiovascular-related chronic disease. Medicine.

[31] Campbell DJ, Manns BJ, Hemmelgarn BR, Sanmartin C, Edwards A, King-Shier K (2017). Understanding financial barriers to care in patients with diabetes. Diabetes Educ..

[32] Campbell DJ, Manns BJ, Hemmelgarn BR, Sanmartin C, King-Shier KM (2016). Development of a conceptual framework for understanding financial barriers to care among patients with cardiovascular-related chronic disease: a protocol for a qualitative (grounded theory) study. CMAJ Open..

[33] Sandelowski M (1995). Sample size in qualitative research. Res Nurs Health.

[34] Ward DR, Novak E, Scott-Douglas N, Brar S, White M, Hemmelgarn BR (2013). Assessment of the Siksika chronic disease nephropathy-prevention clinic. Can Fam Physician.

[35] Aronson J (1995). A pragmatic view of thematic analysis. Qual Rep..

[36] Turner A, Crompton S, Langlois S (2013). Aboriginal peoples in Canada: First Nations People, Métis and Inuit.

[37] Adelson N (2005). The embodiment of inequity: health disparities in aboriginal Canada. Can J Public Health.

[38] Haman F, Fontaine-Bisson B, Batal M, Imbeault P, Blais JM, Robidoux MA (2010). Obesity and type 2 diabetes in Northern Canada’s remote First Nations communities: the dietary dilemma. Int J Obes.

[39] http://www.hc-sc.gc.ca/fniah-spnia/pubs/nihb-ssna/_drug-med/2016-prov-fourn-guide/index-eng.php. Accessed 14 July 2016. HCPgfpbN-iHB.

[40] Ganesh A, King-Shier K, Manns BJ, Hill MD, Campbell DJ (2017). Money is brain: financial barriers and consequences for Canadian stroke patients. Can J Neurol Sci.

[41] Ramsey SD, Newton K, Blough D, McCulloch DK, Sandhu N, Reiber GE (1999). Incidence, outcomes, and cost of foot ulcers in patients with diabetes. Diabetes Care.

[42] Klein R, Klein BE, Moss SE (1996). Relation of glycemic control to diabetic microvascular complications in diabetes mellitus. Ann Intern Med.

[43] Harding JJ, Egerton M, van Heyningen R, Harding RS (1993). Diabetes, glaucoma, sex, and cataract: analysis of combined data from two case control studies. Br J Ophthalmol.

[44] Fledelius HC (1986). Myopia and diabetes mellitus with special reference to adult-onset myopia. Acta Ophthalmol.

[45] Moss SE, Klein R, Klein BE (1988). The incidence of vision loss in a diabetic population. Ophthalmology.

[46] Tessaro I, Smith SL, Rye S (2005). Knowledge and perceptions of diabetes in an Appalachian population. Prev Chronic Dis..

[47] Wilson D, Macdonald D (2010). The income gap between Aboriginal peoples and the rest of Canada.

[48] Shah BR, Gunraj N, Hux JE (2003). Markers of access to and quality of primary care for aboriginal people in Ontario, Canada. Am J Public Health..

[49] Smith KB, Humphreys JS, Wilson MG (2008). Addressing the health disadvantage of rural populations: how does epidemiological evidence inform rural health policies and research?. Aust J Rural Health.

[50] KaLAaBJaAP Adams (2017). How’s your sugar? evaluation of a website for aboriginal people with diabetes. JMIR Diabetes..

[51] Tervalon M, Murray-Garcia J (1998). Cultural humility versus cultural competence: a critical distinction in defining physician training outcomes in multicultural education. J Health Care Poor Underserved.

[52] Campbell D (2012). Cultural competency in Haitian-serving community health centers in South Florida. Mcgill J Med..

[53] Turton CL (1997). Ways of knowing about health: an aboriginal perspective. ANS Adv Nurs Sci..

[54] Morse JM (2000). Determining sample size. Qual Health Res.

[55] AaSJ Rodriguez (2018). Phenomenology as a healthcare research method. Evid Based Nurs..

[56] Boddy CR (2016). Sample size for qualitative research. Qual Mark Res Int J..

[57] Cochran PA, Marshall CA, Garcia-Downing C, Kendall E, Cook D, McCubbin L (2008). Indigenous ways of knowing: implications for participatory research and community. Am J Public Health.

[58] Boston P, Jordan S, MacNamara E, Kozolanka K, Bobbish-Rondeau E, Iserhoff H (1997). Using participatory action research to understand the meanings aboriginal Canadians attribute to the rising incidence of diabetes. Chronic Dis Can..

[59] Bartlett JG, Iwasaki Y, Gottlieb B, Hall D, Mannell R (2007). Framework for Aboriginal-guided decolonizing research involving Metis and First Nations persons with diabetes. Soc Sci Med.

